# Supplementary material for: Ubiquitination of RIPK1 regulates its activation mediated by TNFR1 and TLRs signaling in distinct manners
Source: Nat Commun. 2020 Dec 11;11:6364. doi: 10.1038/s41467-020-19935-y (PMC7733462; doi:10.1038/s41467-020-19935-y)
Supplement: Supplementary file 1 — Supplementary Information [file 41467_2020_19935_MOESM1_ESM.pdf]

Ubiquitination of RIPK1 regulates its activation mediated by TNFR1 and TLR signaling in distinct manner

10/27/2020

Xingyan Li[1], Mengmeng Zhang[1], Xinyue Huang[1], Wei Liang[1], Ganquan Li[1], Xiaojuan Lu[1], Yanxia Li[1], Heling Pan[1], Linyu Shi[1], Hong Zhu[2], Lihui Qian[1], Bing Shan[1]* and Junying Yuan[1]*.

[1]Interdisciplinary Research Center on Biology and Chemistry, Shanghai Institute of Organic Chemistry, Chinese Academy of Sciences, 26 Qiuyue Rd, PuDong District, Shanghai, 201203, China.

[2]Department of Cell Biology, Harvard Medical School, 240 Longwood Ave. Boston, MA 02115. USA.

*Corresponding authors. Emails: shanbing@sioc.ac.cn; junying_yuan@sioc.ac.cn

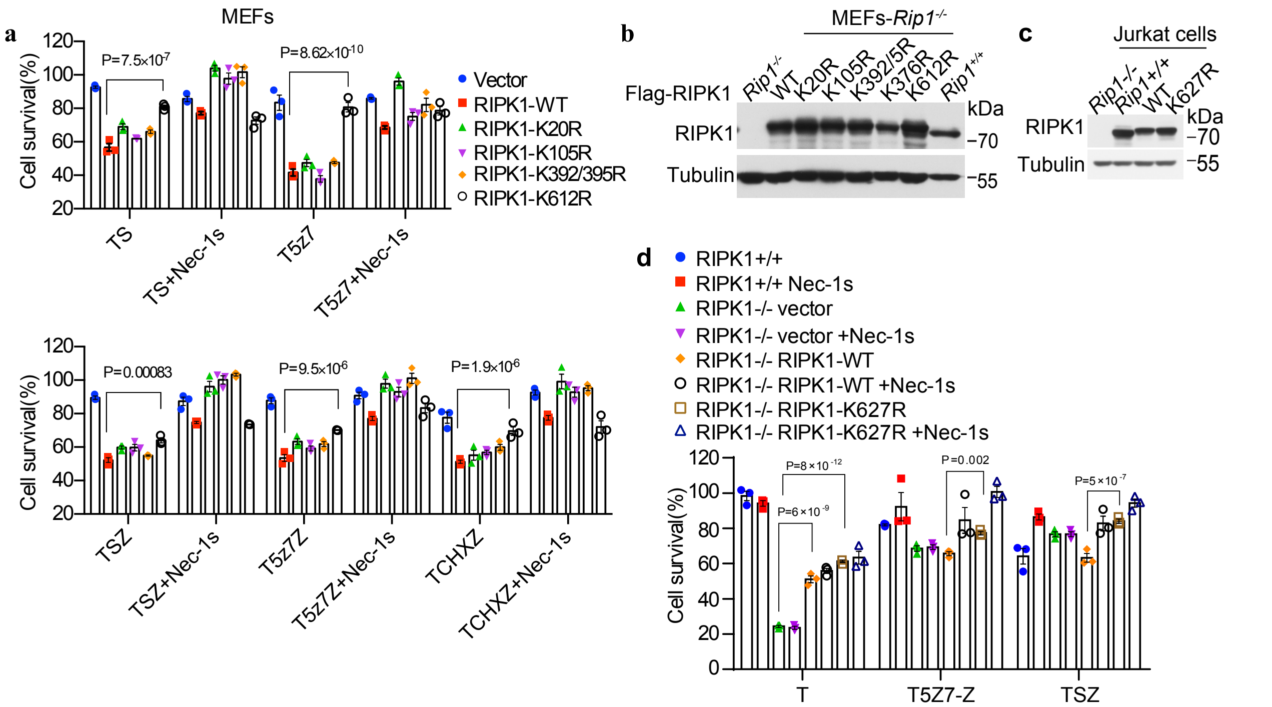

Supplementary Figure 1 K612 in mRIPK1/K627 in hRIPK1 is important for mediating RDA and necroptosis induced by TNFα. (a, b) Ripk1$^{-/-}$ MEFs were reconstituted with WT or lysine mutant RIPK1 using PMSCV retrovirus infection. The reconstituted MEFs were pretreated with SM-164 (100 nM), 5Z-7 (200 nM) or CHX (2 μg/ml) for 2 h as indicated, and then treated with TNFα (100 ng/ml) to induce apoptosis or TNFα (100 ng/ml) plus Z-VAD (25 μM) (a) to induced necroptosis for 12 h. Cell survival was measured by CellTiterGlo. Data are presented as Mean±SEM of n=3 biologically independent samples. Two-way ANOVA with Bonferroni's multiple comparison test. The RIPK1 protein expression levels of indicated MEFs were analyzed by western blotting (b). Uncropped blots in the Source Data file.
(c, d) Jurkat RIPK1 deficient cells were reconstituted with WT or K627R RIPK1 individually using Tet-On® Advanced Inducible Expression System. RIPK1 protein expression levels of indicated Ripk1+/+ and reconstituted Jurkat cells were analyzed by western blotting (c). After induction for 24 h with doxycycline, reconstituted Jurkat cells were pretreated with SM-164 (100 nM) or 5Z-7 (200 nM) for 2 h, and then treated with TNFα (100 ng/ml) plus Z-VAD (25 μM) for about 24 h. Cell survival was measured by CellTiterGlo (d). Data are presented as Mean±SEM of n=3 biologically independent samples. Two-way ANOVA with Bonferroni's multiple comparison test. Uncropped blots in the Source Data file.

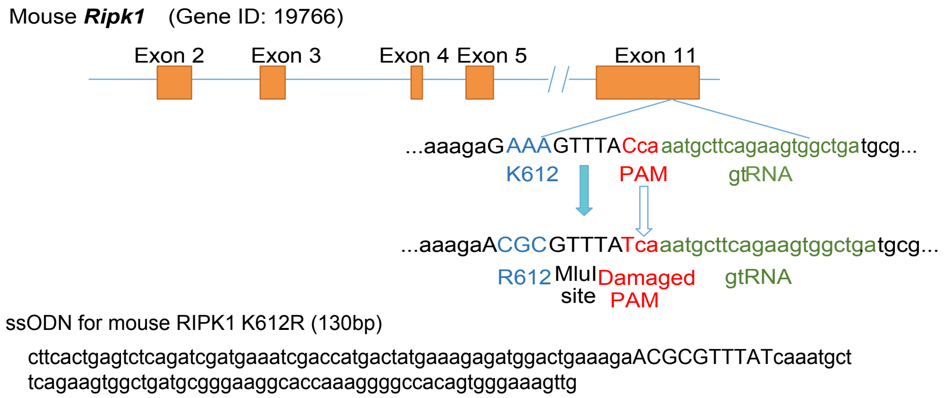

Supplementary Figure 2 Strategy of generating *Ripk1*$^{K612R/K612R}$ mutant mice.
Ripk1$^{K612R/K612R}$ knockin mice were generated by mutating lysine codon-AAA at position 612 to arginine codon-CGC via CRISPR/Cas9 system. The single-guide RNA and template DNA were injected into cytoplasm of B6D2F1 zygotes. Sequence of sgRNA(5'-aatgcttcagaagtggctga-3') and template DNA (5'-cttcactgagtctcagatcgatgaaatcgaccatgactatgaaagag atggactgaaagaacgcgtttatcaaatgcttcagaagtggctgatgcgggaaggcaccaaaggggccacagtgggaaagttg-3'). We also introduced a synonymous mutation at Y614 site (TAC to TAT) to disrupt sgRNA recognize site and generate a Mlu I restriction site.

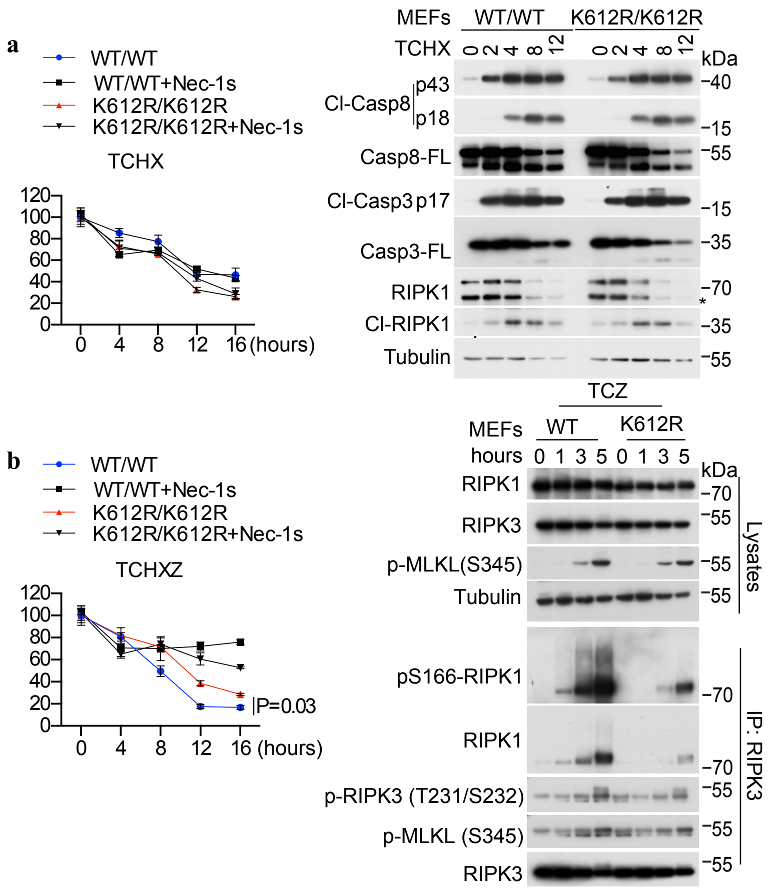

Supplementary Figure 3 Ripk1^{K612R} mutant MEFs did not show any resistance to RIPK1-independent apoptosis. (a,b) *Ripk1*$^{+/+}$ and *Ripk1*$^{K612R/K612R}$ MEFs were pretreated with CHX (2 μg/ml) for 1.5 h in the presence or absence of Nec-1s (10 μM), and then treated with TNFα (100 ng/ml) (a) or TNFα (100 ng/ml) plus Z-VAD (25 μM) for indicated time (b). Cell survival was measured by Cell TiterGlo. Mean±SEM of n=3 biologically independent samples. Two-way ANOVA with Bonferroni's multiple comparison test. The cell lysates were analyzed by western blotting using indicated antibodies (a,b). Uncropped blots in Source Data file.

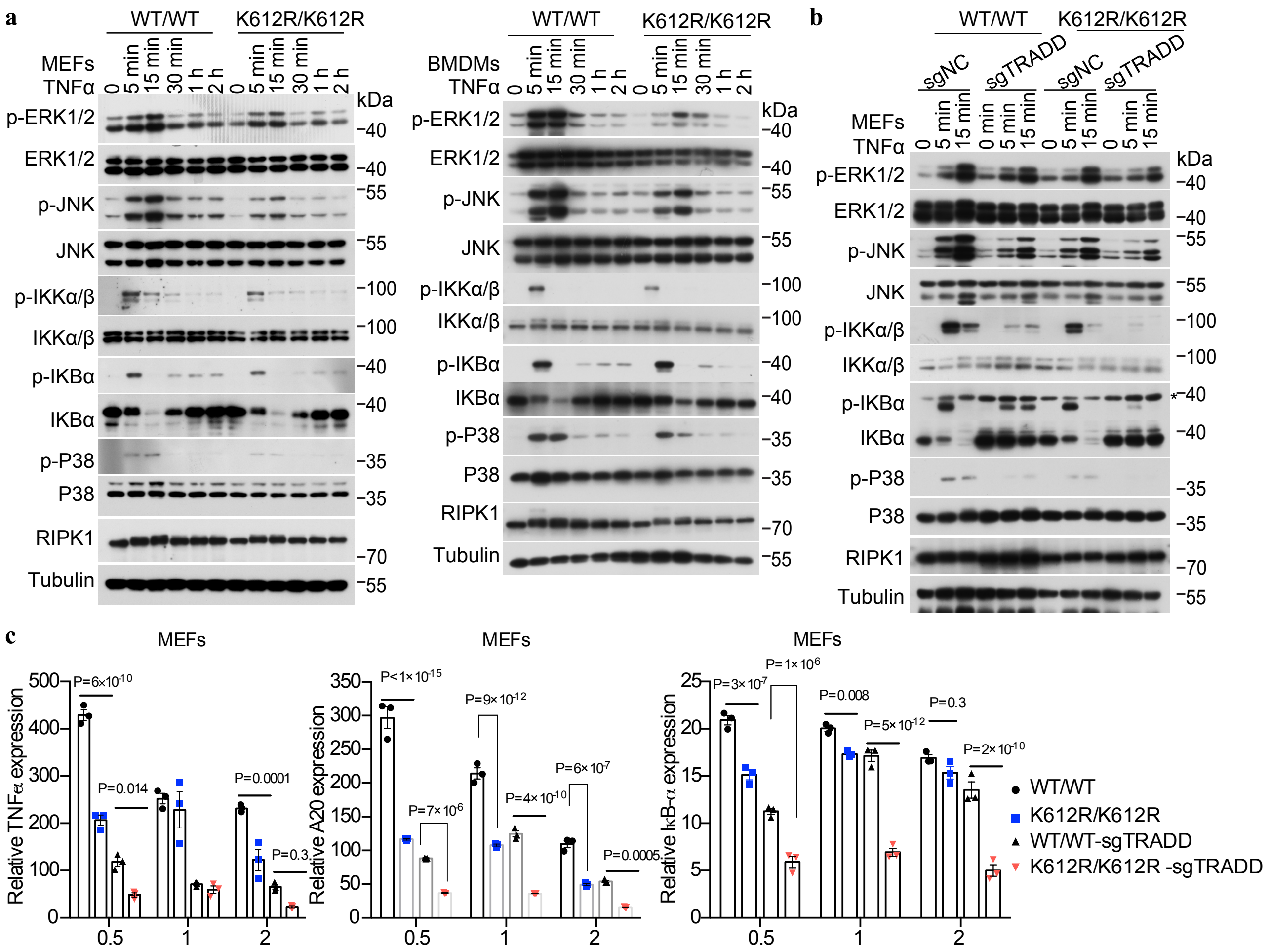

Supplementary Figure 4 RIPK ^{K612R} mutation impairs the activation of NF-κB and MAPK. (a)*Ripk1*$^{+/+}$, *Ripk1*$^{K612R/K612R}$ MEFs and primary BMDMs were treated with mTNFα (100 ng/ml) for indicated time points. The cells were lysed in SDS reducing sample buffer. Cell lysates were analyzed by western blotting using indicated antibodies. Uncropped blots in the Source Data file.(b, c) TRADD was deleted in WT and K612R mutant MEF cells by CRISPR/Cas9. MEFs were treated with mTNFα (100 ng/ml) for indicated time points. Cells were lysed with SDS reducing sample buffer. Cell lysates were analyzed by western blotting using indicated antibodies (b). Cells were lysedin TRIzol. RNAs were extracted and qRT-PCR was performed to analyze cytokine transcription levels (c). Data are presented as Mean±SEM of n=3 biologically independent samples. Two-way ANOVA with Bonferroni's multiple comparison test.

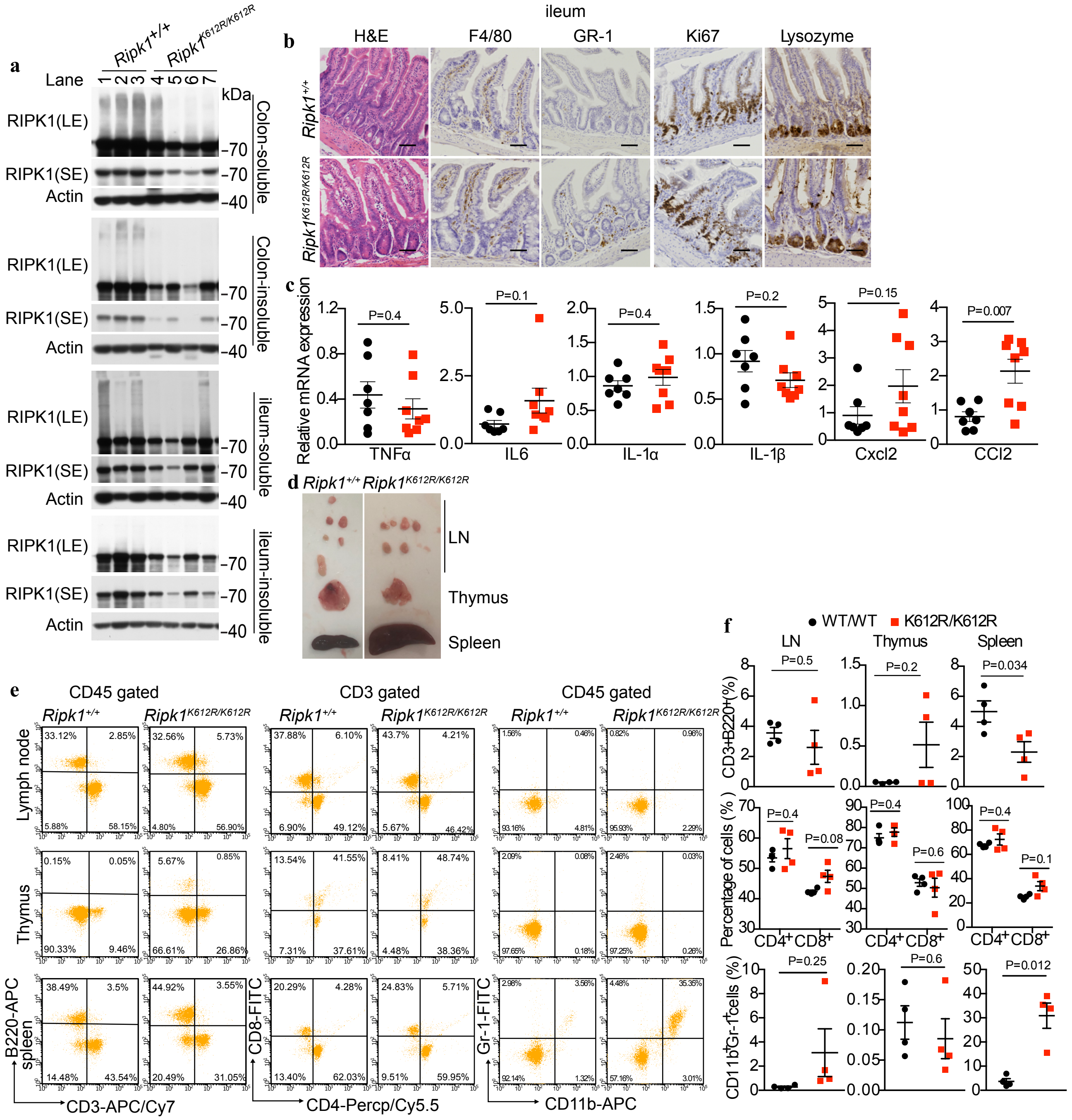

Supplementary Figure 5 *Ripk1*$^{K612R/K612R}$ mice develop adult-onset intestinal inflammation and splenomegaly. (a) Western blotting analysis of colon and ileum from 20 weeks-old *Ripk1*$^{+/+}$ (n=3) and *Ripk1*$^{K612R/K612R}$ mice (n=4) for RIPK1 levels and actin was a loading control. LE: light exposure; SE: strong exposure. Uncropped blots in the Source Data file. (b) H&E staining and immunohistochemical staining of F4/80, GR-1, Ki67 and lysozyme in the sections of ileum from 8-9 weeks-old *Ripk1*$^{+/+}$ (n=4) and *Ripk1*$^{K612R/K612R}$ (n=4) mice (Scale bars, 50μm). (c) qRT-PCR analysis of cytokines and chemokines expression in the ileum of 8-weeks-old *Ripk1*$^{+/+}$ (n=7) and *Ripk1*$^{K612R/K612R}$ (n=8) mice. Data are presented as Mean±SEM of n=8 biologically independent mice. Unpaired two-tailed Student's t test. (d) Representative images of lymph nodes, thymus and spleens of 20 weeks-old *Ripk1*$^{+/+}$and *Ripk1*$^{K612R/K612R}$ mice.(e-f) FCAS analysis for the populations of CD3^{+}B220^{+}, CD4^{+}, CD8^{+} and CD11b^{+}GR-1^{+} cells in lymph nodes thymus and spleens about 20 weeks-old *Ripk1*$^{+/+}$ (n=4) and *Ripk1*$^{K612R/K612R}$ (n=4) mice.Data are presented as Mean±SEM of n=4 biologically independent mice. Unpaired two-tailed Student's t test.

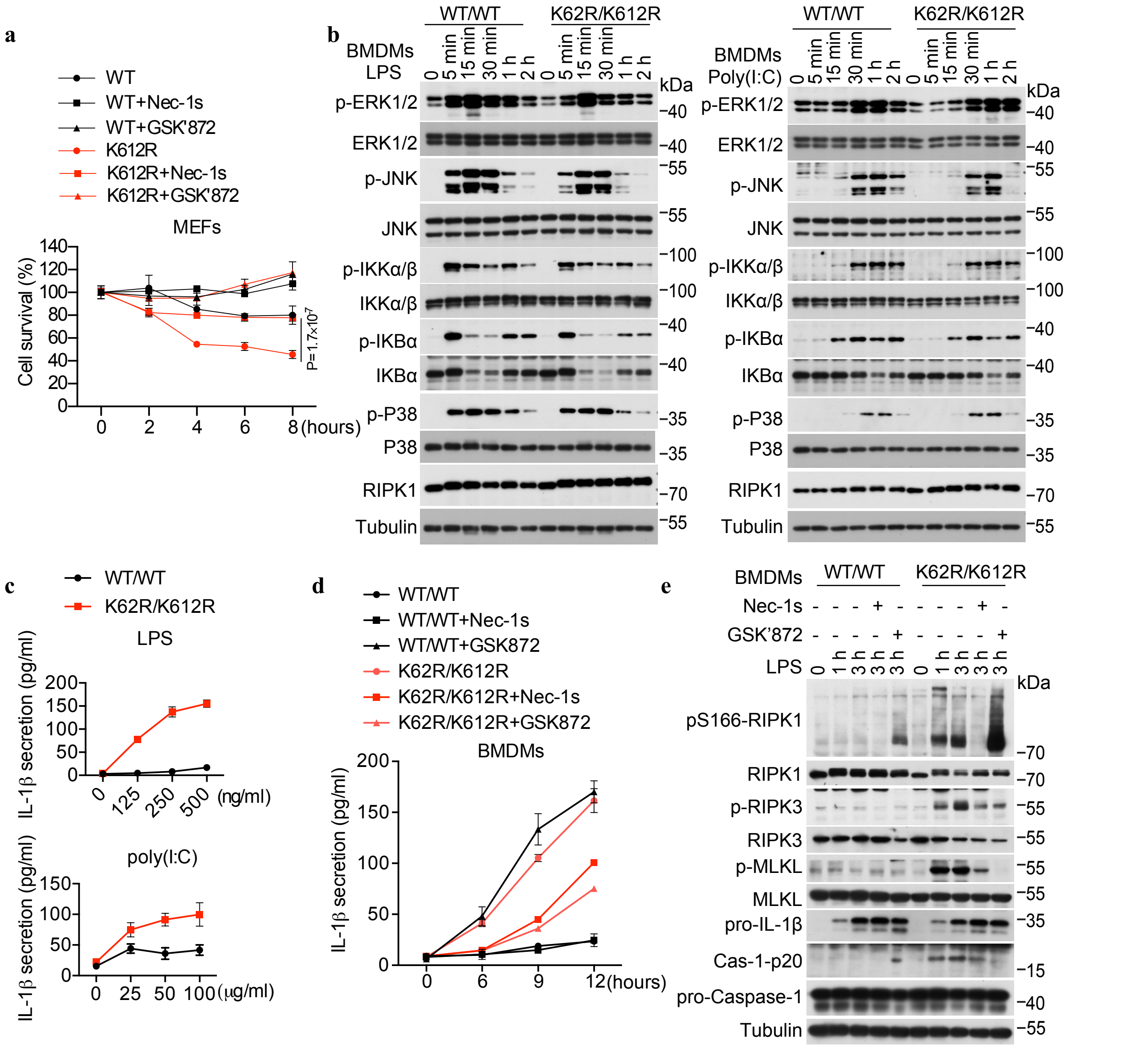

Supplementary Figure 6 Ripk1^{K612R}mutation promotes necroptosis and caspase-1 activation mediated by TLR3/4. (a) *Ripk1*$^{+/+}$ and *Ripk1*$^{K612R/K612R}$ MEFs were pretreated with Nec-1s (10 μM), GSK'872 (10 μM) or vehicle in the presence or absence of Z-VAD (25 μM) for 30 min as indicated, and then treated with Poly (I:C) (20 μg/ml) as indicated for 12 h. Cell survival was measured by Cell TiterGlo. Mean±SEM of n=3 biologically independent samples. Two-way ANOVA with Bonferroni's multiple comparison test. (b) *Ripk1*$^{+/+}$ and *Ripk1*$^{K612R/K612R}$ primary BMDMs were treated with LPS (50 ng/ml) or Poly (I:C) (20 μg/ml) for indicated time points. The cells were lysed in SDS reducing sample buffer. Cell lysates were analyzed by western blotting using indicated antibodies. (c) Quantitation of IL-1β in the cultural supernatant of *Ripk1*$^{+/+}$ and *Ripk1*$^{K612R/K612R}$ primary BMDMs treated with Poly (I:C) (20 μg/ml) or LPS (50 ng/ml) for 12 h as indicated by ELISA. (d, e) Quantitation of IL-1β in the cultural supernatant by ELISA (d) or western blotting analysis (e) of *Ripk1*$^{+/+}$ and *Ripk1*$^{K612R/K612R}$primary BMDMs pretreated with Nec-1s (10 μM) or GSK'872 (10 μM) for 30 min, and then treated with LPS (50 ng/ml) for indicated time points. Uncropped blots in the Source Data file.

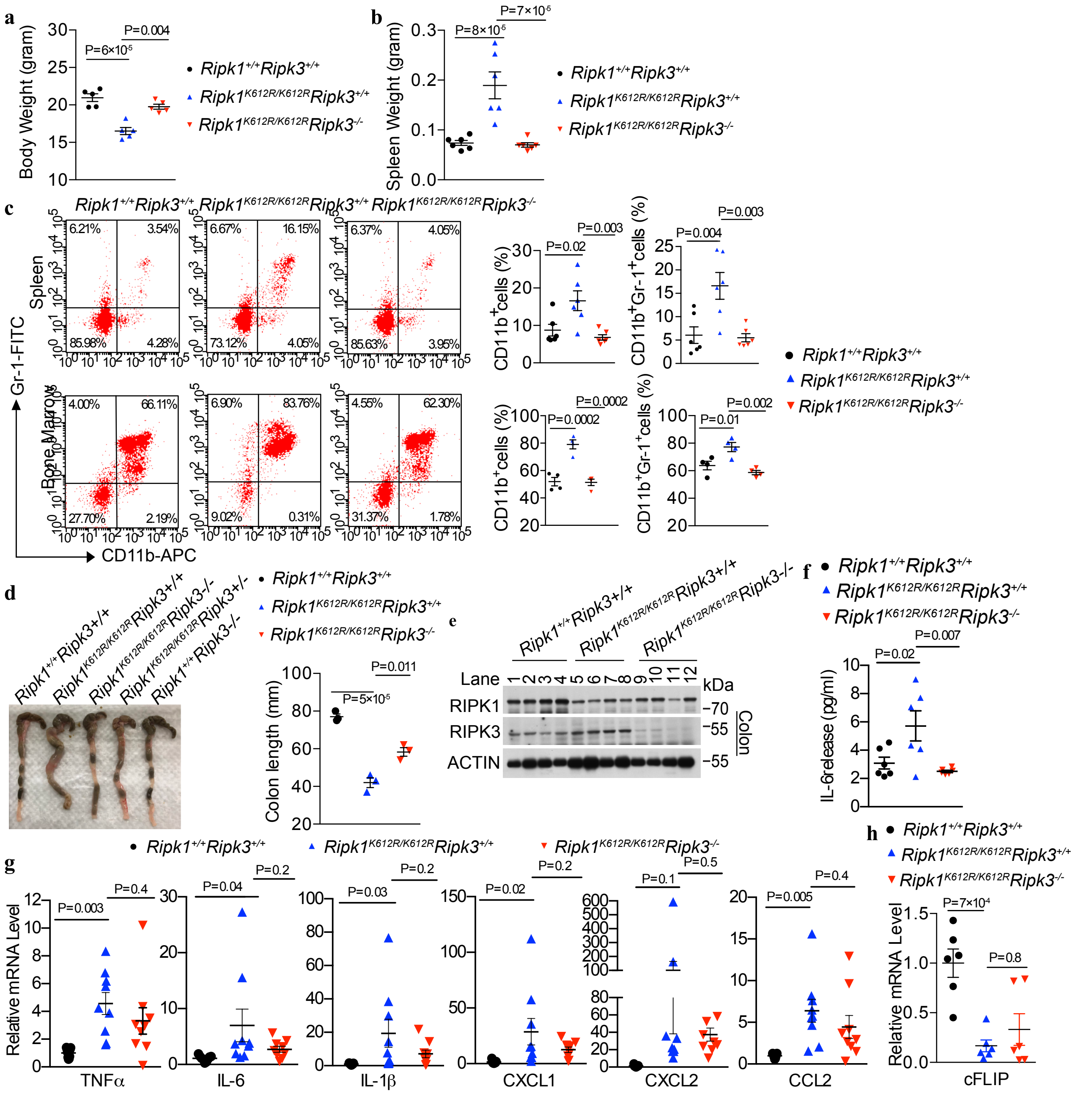

Supplementary Figure 7 RIPK3 deficiency ameliorates gut inflammation and prevents splenomegaly of *Ripk1*$^{K612R/K612R}$ mice. (a) Body weight of male *Ripk1*$^{+/+}$*Ripk3*$^{+/+}$ (n=5), *Ripk1*$^{K612R/K612R}$ *Ripk3*$^{+/+}$ (n=5) and *Ripk1*$^{K612R/K612R}$ *Ripk3*$^{-/-}$mice (n=5) at 8 weeks of age. Data are presented as Mean±SEM of n=5 biologically independent mice. One-way ANOVA with Dunnett's multiple comparison test. (b) Spleen weight of 8-weeks-old *Ripk1*$^{+/+}$*Ripk3*$^{+/+}$ (n=6), *Ripk1*$^{K612R/K612R}$ *Ripk3*$^{+/+}$ (n=6) and *Ripk1*$^{K612R/K612R}$ *Ripk3*$^{-/-}$mice (n=6). Data are presented as Mean±SEM of n=6 biologically independent mice. One-way ANOVA with Dunnett's multiple comparison test. (c) FCAS analysis for the populations of CD45^{+}CD11b^{+}Gr-1^{+}cells in the spleens of 8-weeks-old *Ripk1*$^{+/+}$*Ripk3*$^{+/+}$ (n=6), *Ripk1*$^{K612R/K612R}$ *Ripk3*$^{+/+}$ (n=6) and *Ripk1*$^{K612R/K612R}$ *Ripk3*$^{-/-}$ mice (n=6) and bone marrow of 8-weeks-old *Ripk1*$^{+/+}$*Ripk3*$^{+/+}$ (n=4), *Ripk1*$^{K612R/K612R}$ *Ripk3*$^{+/+}$(n=4) and *Ripk1*$^{K612R/K612R}$ *Ripk3*$^{-/-}$mice (n=4). Dot plot indicated the percentages of indicated cell populations. Data are presented as Mean±SEM of n=6 or 4 biologically independent mice. One-way ANOVA with Dunnett's multiple comparison test.(d) Representative images of large intestines, colon length of male *Ripk1*$^{+/+}$*Ripk3*$^{+/+}$ (n=3), *Ripk1*$^{K612R/K612R}$ *Ripk3*$^{+/+}$(n=3) and *Ripk1*$^{K612R/K612R}$ *Ripk3*$^{-/-}$mice (n=3) at 8 weeks of age. (e) RIPK1 and RIPK3 protein levels of colon sections from indicated mice were analyzed by western blotting using indicated antibodies. Uncropped blots in the Source Data file. (f) Quantitation of IL-6 in the sera of 8-weeks-old *Ripk1*$^{+/+}$*Ripk3*$^{+/+}$ (n=6), *Ripk1*$^{K612R/K612R}$ *Ripk3*$^{+/+}$ (n=6) and *Ripk1*$^{K612R/K612R}$ *Ripk3*$^{-/-}$mice (n=6) by ELISA. Data are presented as Mean±SEM of n=6 biologically independent mice. One-way ANOVA with Dunnett's multiple comparison test. (g) QRT-PCR analysis of cytokine and chemokine in the colon of 8-weeks-old *Ripk1*$^{+/+}$*Ripk3*$^{+/+}$ (n=9), *Ripk1*$^{K612R/K612R}$ *Ripk3*$^{+/+}$ (n=9) and *Ripk1*$^{K612R/K612R}$ *Ripk3*$^{-/-}$mice (n=9). Data are presented as Mean±SEM of n=9 biologically independent mice. One-way ANOVA with Dunnett's multiple comparison test.(h) QRT-PCR analysis of cFLIP expression in the colon of 8-weeks-old *Ripk1*$^{+/+}$*Ripk3*$^{+/+}$ mice (n=6), *Ripk1*$^{K612R/K612R}$ *Ripk3*$^{+/+}$mice (n=6) and *Ripk1*$^{K612R/K612R}$ *Ripk3*$^{-/-}$mice (n=6). Data are presented as Mean±SEM of n=6 biologically independent mice. One-way ANOVA with Dunnett's multiple comparison test.

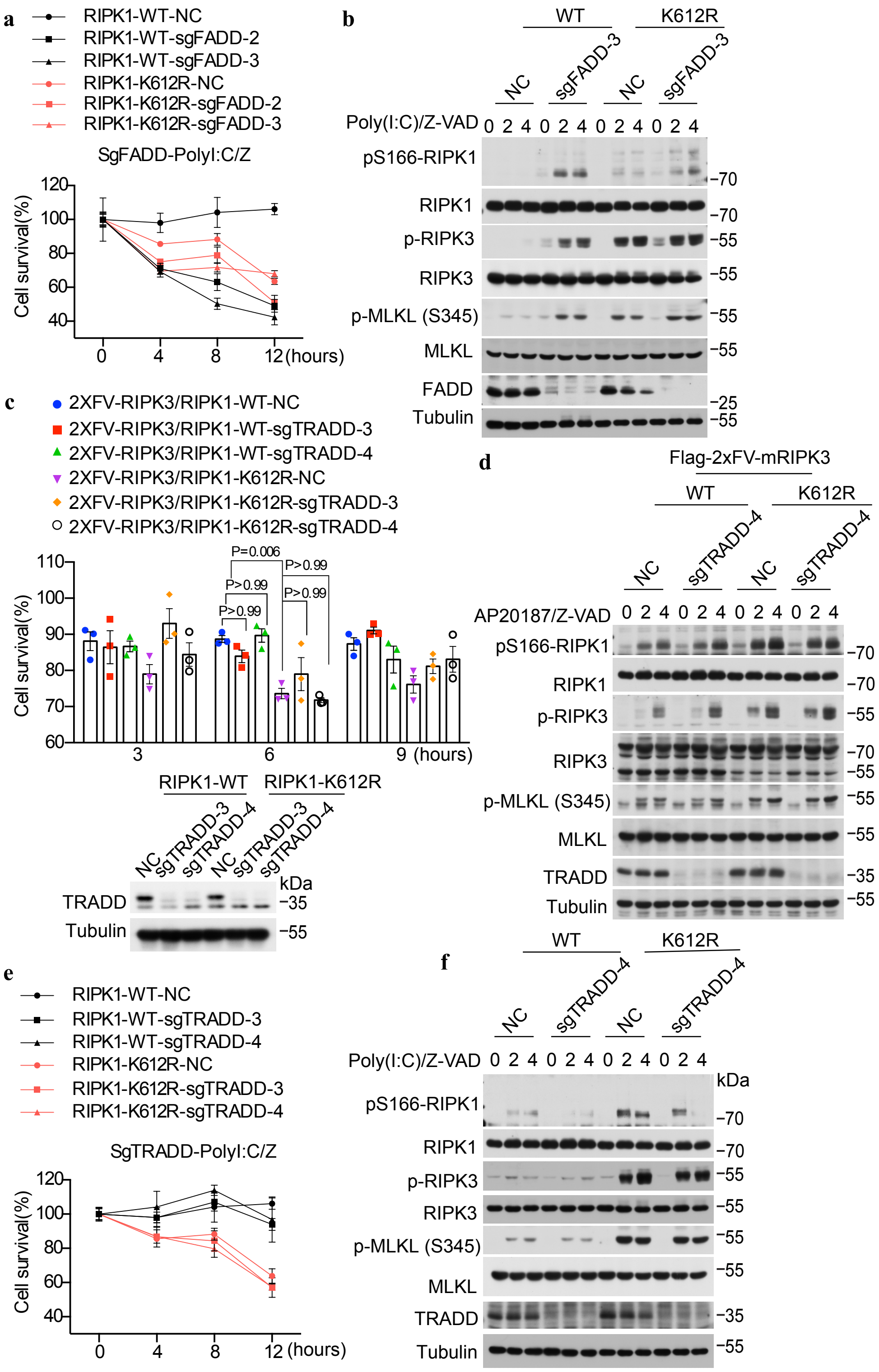

Supplementary Figure 8 FADD but not TRADD is required for RIPK1 K612 to inhibit necroptosis induced by RIPK3. dimerization. (a,b) FADD knockout MEFs were made by CRISPR/Cas9. The cells were treated with Poly (I:C) (20 µg/ml) plus Z-VAD (25 µM) for indicated time. Cell survival was measured by Cell TiterGlo (a). Cells were lysed in SDS reducing sample buffer. Protein levels were detected by western blotting using indicated antibodies (b). Uncropped blots in the Source Data file. (c, d) TRADD knockout MEFs were made by CRISPR/Cas9. The cell lines were made to stably expressing 2xFV-RIPK3 and treated with AP20187 (30 nM) plus Z-VAD (25 µM) for indicated time. Cell survival was measured by Cell TiterGlo (c). Data are presented as Mean±SEM of n=3 biologically independent samples. Two-way ANOVA with Bonferroni's multiple comparison test. The cells were lysed in SDS reducing sample buffer and analyzed by western blotting (d). Uncropped blots in the Source Data file. (e, f) TRADD knockout MEFs were treated with Poly (I: C) (20 µg/ml) plus Z-VAD (25 µM) for indicated time. Cell survival was measured by CellTiterGlo (e). The cells were lysed in SDS reducing sample buffer and analyzed by western blotting(f). Uncropped blots in the Source Data file.

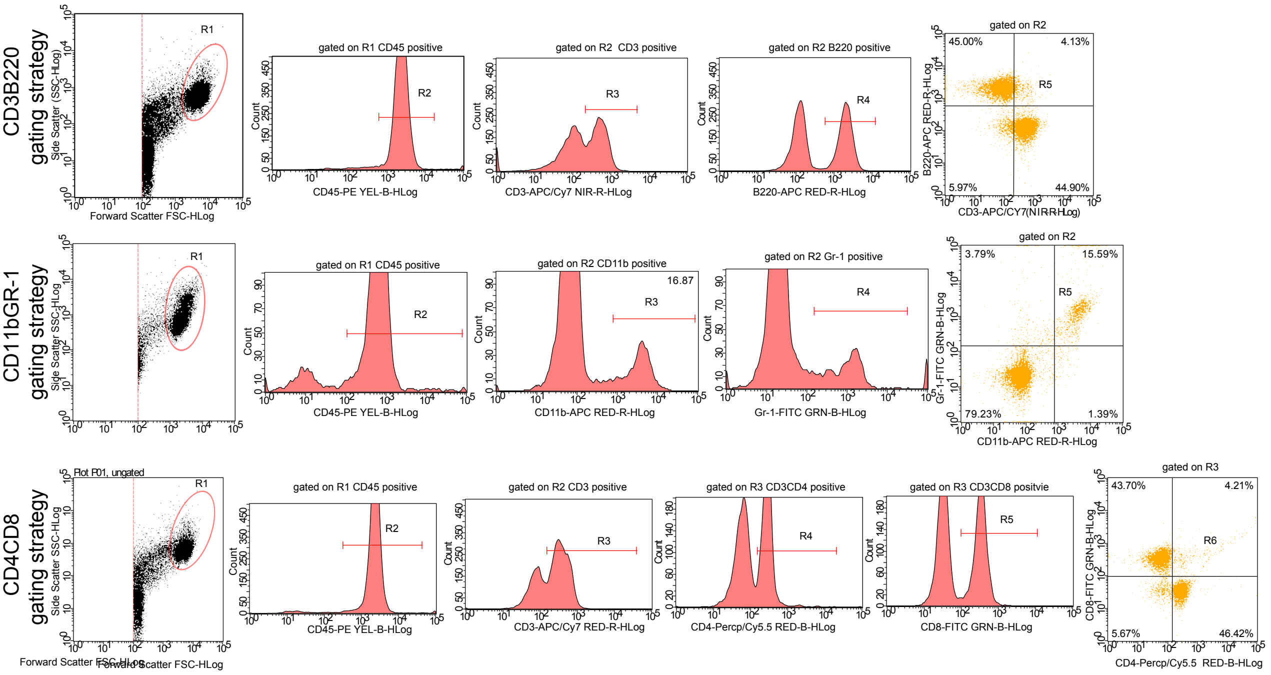

Supplementary Figure 9 Gating strategy for FACS. Dot-plots and Histogram from FACS analysis to illustrate the gating to obtain the different CD3$^+$B220$^+$, CD11b$^+$GR-1$^+$ and CD3$^+$CD4$^+$, CD3$^+$CD8$^+$ subpopulations in thymus, spleen and Lymph nods.

Ubi site	Localization prob	Intensity_K627R	Intensity_WT	Ratio_WT/K627R
13	1	5.19E+07	7.59E+07	0.6834
30	1	5.15E+06	1.29E+07	0.3976
45	0.9964	1.45E+06	2.51E+06	0.5774
49	0.9995	1.75E+06	3.65E+06	0.4786
65	1	6.88E+05	1.70E+06	0.4053
87	1	7.37E+06	1.24E+07	0.5930
97	0.9999	6.69E+05	2.92E+06	0.2292
105	1	2.05E+06	2.32E+06	0.8825
115	1	1.52E+08	1.33E+08	1.1466
132	0.8898	1.07E+06	2.47E+06	0.4339
137	1	2.08E+07	3.56E+07	0.5843
140	1	3.26E+07	6.30E+07	0.5178
153	0.9998	5.86E+05	2.24E+06	0.2610
163	1	2.31E+07	1.03E+08	0.2245
167	1	1.41E+07	5.98E+07	0.2363
185	1	1.07E+07	1.06E+07	1.0106
204	0.9843	2.74E+06	2.90E+06	0.9457
265	1	0	6.15E+05	0
284	1	3.91E+06	1.01E+07	0.3889
302	0.8296	0	5.60E+07	0
306	1	1.41E+08	2.46E+08	0.5733336
316	1	4.72E+07	9.34E+07	0.5050287
565	0.9999	6.48E+07	9.78E+07	0.6623322
571	1	1.29E+07	2.65E+07	0.4879453
585	1	6.01E+07	5.63E+07	1.0686
596	1	8.02E+07	1.96E+07	4.0975
604	1	2.56E+08	1.20E+08	2.1371
627	1	0	2.68E+07	0
634	1	8.30E+07	9.15E+06	9.0713
642	1	1.34E+09	1.29E+09	1.0331
648	1	4.68E+07	1.43E+07	3.2671

Supplementary Table 1. Quantitative mass spectrometry analysis of ubiquitination sites in human RIPK1 overexpressed in 293T cells. HEK293T cells were transfected with expression vectors of flag-hRIPK1-WT or flag-hRIPK1-K627R for about 18 h. Cell lysates were immunoprecipitated with Flag M2 Agarose Affinity gel. Flag-RIPK1 was trypsin-digested and subjected to enrichment of diGly peptides. The ubiquitination sites with localization lower than 75% were excluded in further analysis. The intensity of each ubiquitinated site (K) were normalized by the RIPK1 level and the ratios of each ubiquitinated site (K) in K627R RIPK1/WT RIPK were shown in the table.

Ubi site	Localization prob	Intensity WT_T0	Intensity WT_T5	Intensity K612R_T0	Intensity K612R_T5	K612R_T0 /WT_T0	K612R_T5/ WT_T5	WT_ T5/T0
20	1	1.16E+07	8.34E+07	5.17E+06	5.24E+07	0.4460	0.6284	7.1971
30	1	1.49E+07	8.45E+06	4.64E+06	7.69E+06	0.3108	0.9092	0.5668
45	0.9640	8.46E+05	1.88E+06	7.22E+05	8.79E+05	0.8524	0.4676	2.2205
46	1	2.23E+07	2.64E+07	7.70E+06	2.18E+07	0.3455	0.8269	1.1832
65	1	1.37E+06	3.52E+06	1.29E+06	1.94E+06	0.9408	0.5507	2.5766
105	1	0	2.83E+08	7.45E+06	8.96E+07	N.D.	0.3168	N.D.
115	1	1.64E+08	0	1.12E+08	1.16E+08	0.6825	N.D.	0
137	1	7.53E+06	9.50E+06	1.92E+06	0	0.2549	0	1.2622
140	1	8.77E+06	1.64E+07	7.25E+06	1.21E+07	0.8268	0.7381	1.8720
153	1	3.69E+06	7.06E+06	3.02E+06	5.03E+06	0.8181	0.7133	1.9143
163	0.9998	1.65E+07	4.87E+07	8.67E+06	2.20E+07	0.5264	0.4523	2.9588
167	1	3.35E+07	4.50E+07	1.03E+07	3.28E+07	0.3060	0.7278	1.3427
307	1	2.09E+08	0	1.27E+08	4.31E+06	0.6060	N.D..	0
376	1	5.98E+07	1.67E+09	6.69E+07	5.76E+08	1.1192	0.3462	27.8415
392	1	1.16E+07	6.27E+07	5.05E+06	3.30E+07	0.4367	0.5255	5.4205
395	1	9.78E+05	1.99E+07	0	1.38E+07	0	0.6941	20.3303
429	1	7.00E+07	5.52E+08	8.60E+06	1.76E+08	0.1228	0.3187	7.8813
550	1	0	2.21E+07	2.09E+06	0	N.D.	0	N.D.
612	1	1.74E+06	2.30E+06	0	0	0	0	1.3232
619	1	0	2.52E+06	1.76E+06	0	0	0	N.D.
627	1	2.85E+08	0	9.61E+07	6.66E+08	0.3368	N.D.	0
633	1	4.64E+06	5.07E+06	6.33E+06	7.43E+06	1.3655	1.4652	1.0932

Supplementary Table 2. Quantitative mass spectrometry analysis of ubiquitination sites in endogenous RIPK1 from mouse embryonic fibroblasts. *Ripk1*$^{+/+}$ and *Ripk1*$^{K612R/K612R}$ immortalized MEFs were without (T0) or with mTNFα (100 ng/ml) for 5 mins (T5). Endogenous RIPK1 was immunoprecipitated with a rabbit monoclonal anti-RIPK1 antibody and then the ubiquitination pattern of endogenous RIPK1 were analyzed by quantitative mass spectrometry. The ubiquitination sites with localization lower than 75% were excluded in further analysis. The intensity of each ubiquitinated site (K) were normalized by the RIPK1 level and the ratios of each ubiquitinated site (K) were shown in the table (N.D.: not determined).

Primers for murine inflammatory cytokines		
Gene	Forward(5'- -3')	Reverse(5'- -3')
TNFα	ACCCTGGTATGAGCCCATATAC	ACACCC ATTCCCTTCACAGAG
Il6	TACCACTCCCAACAGACCTG	GGTACTCCAGAAGACCAGAGG
Il-1α	CGCTTGAGTCGGCAAAGAAAT	TGGCAGAACTGTAGTCTTCGT
Il-1β	ACTCATTGTGGCTGTGGAGA	TTGTTCATCTCGGAGCCTGT
Cxcl1	CTATCGCCAATGAGCTGCG	CTATCGCCAATGAGCTGCG
Cxcl2	CTCTCAAGGGCGGTCAAAAAGTT	TCAGACAGCGAGGCACATCAGGTA
Ccl2	AGCTGTAGTTTTTGTCACCAAGC	GACCTTAGGGCAGATGCAGT
IκBα	TGACTTTGGGTGCTGATGTC	AAGCTGGTAGGGGGAGTAGC
cFlip	GCTCCAGAATGGGCGAAGTAA	ACGGATGTGCGGAGGTAAAAA
A20	ACCCCAGCTATCACTCATGG	CTCTGAGCATTCGTGGCATA
β-Actin	CAGCCTTCCTTCTTGGGTAT	GGTCTTTACGGATGTCAACG
Primers for murine gene deletion		
Gene(sg)	Forward(5'- -3')	Reverse(5'- -3')
Fadd(sg-2)	CACCGCCATCAGATCGTTGCCCGAC	AAACGTCGGGCAACGATCTGATGGC
Fadd(sg-3)	CACCGCCTGTCGGGCAACGATCTGA	AAACTCAGATCGTTGCCCGACAGGC
Tradd(sg-3)	CACCGTTCCTCCAAGCCTACCGCGA	AAACTCGCGGTAGGCTTGGAGGAAC
Tradd(sg-4)	CACCGTCGTCCAGTTGCGGTTCTGC	AAACGCAGAACCGCAACTGGACGAC
Primers for genotyping		
Gene	Forward(5'- -3')	Reverse(5'- -3')
$Ripk1^{K612R}$	CCTGGGCCTTTTCAGCAA	AAACGGGGTTTGTGCACCAG
$Ripk3^{+/+}$	ATTTGAAAGGGAAGTTGTGAAGTCGCTCAT	CTCCTTTACCCACAAACTCCAGCTTCTTC
$Ripk3^{-/-}$	ACATGCATGGTCATGCACACACAT	GTCGAGGGACCTAATAACTTCGTA

Supplementary Table 3 Primers for murine inflammatory cytokine detection, gene deletion and genotyping.
